# Diet Quality through Adolescence and Early Adulthood: Cross-Sectional Associations of the Dietary Approaches to Stop Hypertension Diet Index and Component Food Groups with Age

**DOI:** 10.3390/nu10111585

**Published:** 2018-10-29

**Authors:** Eleanor M. Winpenny, Stephanie Greenslade, Kirsten Corder, Esther M. F. van Sluijs

**Affiliations:** Centre for Diet and Activity Research & MRC Epidemiology Unit, School of Clinical Medicine, University of Cambridge, Cambridge CB2 1TN, UK; stephanie@greensladeshome.co.uk (S.G.); klc29@medschl.cam.ac.uk (K.C.); ev234@medschl.cam.ac.uk (E.M.F.v.S.)

**Keywords:** diet quality, adolescence, early adulthood, vegetables, fruit, dairy, age

## Abstract

Late adolescence to early adulthood is the period of life when prevalence of overweight and obesity rises the fastest, and an important time to understand changes in dietary risk factors. In this study we assess the variation in diet quality through analysis of cross-sectional data from 2957 individuals aged 13 to 30 from the National Diet and Nutrition Study (2008–2016). Diet data were self-reported using four-day food diaries and coded to give diet quality using the dietary approaches to stop hypertension (DASH) index (range 0–80) and DASH component food groups (grams/day). The mean DASH index score was low at 34.8 (95% confidence interval (CI) = 34.3–35.4). The regression of diet quality score and food groups on age categories revealed no significant change in diet quality score with age category in males, but an improved diet quality score among females aged 19–21 (*β* = 2.04, 95% CI = 0.05–4.02), 25–27 (*β* = 3.77, 95% CI = 1.36–6.18) and 28–30 (*β* = 2.39, 95% CI = 0.53–4.26), compared to age 13–15. Both sexes showed increased vegetable intake with age. Dairy intake was lower in early adult ages among males, while in females there was an increase in the proportion of low-fat dairy consumed with age. Further research should address the determinants of changes in diet in early adulthood to provide evidence for the targeting of public health policy.

## 1. Introduction

Poor quality diet in adulthood increases the risk of obesity and chronic disease (e.g., diabetes, cardio-vascular disease, and certain cancers) [1]. The period of life from late adolescence to early adulthood is the time when the prevalence of being overweight and obesity rises the fastest [2], and an important time for understanding changes in determinants of obesity such as diet and physical activity. It is also a time when individuals go through many life transitions likely to be associated with changes in the determinants of dietary behaviors [3]. Better understanding of how diet changes across this age range will lay a foundation for further investigation of the determinants of changes in diet, and evidence on how and when best to intervene to promote the establishment of a high-quality diet that persists in adulthood. 

Few studies have analyzed changes in diet through late adolescence and early adulthood [4]. Previous studies reporting data from the National Diet and Nutrition Survey, a cross-sectional survey representative of the national population, have suggested that U.K. adolescents have a poor quality of diet when compared to adolescents from other European countries [5], with 40% of total energy intake derived from non-core foods [6] and 15% of total energy derived from free sugars [7]. However, there have been no studies to date looking at how diet quality varies with age through adolescence and young adulthood in the U.K. population. In the U.S., analysis of the longitudinal NEXT Plus study similarly showed low diet quality among adolescents compared to recommendations, with limited changes in diet quality from age 16 to 20. This included no significant change with age according to a diet quality score, the Healthy Eating Index-2010, a small increase in energy derived from whole plant foods, and a small decrease in empty calories (proportion of energy intake from added sugars, discretionary solid fat, and excess alcohol) consumed [8]. 

Assessment of the health-related quality of diet can be achieved using one of a number of diet quality indices that score diets based on the food and/or nutrient components thought to be most relevant for health outcomes [1,9]. Examples include the Healthy Eating Index, the Mediterranean Diet Score and the Dietary Approaches to Stop Hypertension (DASH) diet index. All indices show similar associations with decreased risk of all-cause, cardiovascular disease (CVD), and cancer mortality [10]. The DASH diet index is based on The Dietary Approaches to Stop Hypertension (DASH) Eating Plan which was initially shown to reduce blood pressure in clinical trials [11]. DASH indices have been associated with reduced risks of mortality related to a wide range of chronic diseases [10] as well as reduced risk of high blood pressure [12] and reduced incidence of metabolic syndrome in adolescence [13]. Inclusion of dairy as a positive component of diet quality [14], and no positive score attributed to moderate levels of alcohol consumption (as in the Mediterranean Diet Score [10]) make this score particularly appropriate for use in adolescents.

Our aim in the present study was to assess cross-sectional associations between diet quality (DASH index [9,15]) and age among adolescents and young adults in the U.K. population. We assess associations between the components of the DASH index and age, to describe the variation in diet with age in the U.K. population, and understand how differences in the intake of particular food groups with age influences overall diet quality. This analysis will provide a foundation for further study of the determinants of changes in diet across this transitional life stage.

## 2. Materials and Methods 

### 2.1. Survey Design and Participants

These analyses comprise secondary analysis of data from years 1 to 8 of the National Diet and Nutrition Survey (NDNS) Rolling Programme (2008–2016), an annual cross-sectional survey that assesses the diet, nutrient intake, and nutritional status of the general population of the U.K. The NDNS aims to recruit 1000 participants each year, comprising an equal ratio of adults (aged 19 years and older) and children (aged 1.5 to 18 years). Households were sampled from the U.K. Postcode Address File, a list of all addresses in the U.K., with up to one adult and one child (18 months or older) from each household eligible for inclusion in the survey [16]. This sampling strategy meant that 2 survey participants could come from the same household, and in the sample used in our analysis, 132 individuals were included from 66 households, however due to the low number (<0.5% of included participants) this was not accounted for in our analysis. Written informed consent was obtained from participants or their parents/guardians. Ethical approval for the NDNS was obtained from the Oxfordshire A Research Ethics Committee and the Cambridge South NRES Committee (Ref. No. 13/EE/0016). In this analysis, we use data on participants aged from 13 to 30 years, from the first eight waves of the NDNS Rolling Programme combined, allowing a sufficiently large sample to analyze associations within an age-based subpopulation. 

### 2.2. Dietary Assessment 

Survey participants were asked to complete a food diary, covering four consecutive days, providing detailed descriptions of each item consumed, time of consumption and estimated amount, based on household measures and photographs, as described previously [17]. Three interview visits per participant were used to: (1) explain the use of the diary prior to completion, (2) to check compliance, deal with any problems, and review the diary to identify and edit possible omissions and missing detail during diary completion, and (3) to review the diary and edit possible omissions and collect the diary following completion. Children aged 12 and older were asked to complete the food diary themselves, but details were confirmed with others where necessary. A food photograph atlas specifically designed for use with young people, was used to assist children with portion size estimation [17]. The protocol was designed so that all days were equally represented across the sample. Data from completed diet diaries were processed by trained diet coders, using the DINO (data in, nutrients out) dietary assessment system [18]. Data files reported food group, nutrient, and energy intake data for each individual, and included weights to adjust for sampling and non-response biases. 

Participants who had completed a food diary over three or more days were eligible for inclusion in the analysis. Individuals reporting consumption of less than 500 kcal/day or greater than 4800 kcal/day were excluded due to implausible energy intake, following an adaption of adult recommendations [19] to take into account the additional energy needs of growing adolescents [20]. 

### 2.3. Processing of Diet Data

Diet quality was assessed using a DASH index, following the methodology used by Gunther et al. [15]. This index provides a diet quality score based on the absolute intake of eight food groups, rather than relative intake within a population, and as such is appropriate for comparison of diet quality across different age groups. Individual data were first adjusted to a total energy intake of 2000 kcal per day using the residual method to account for misreporting of total energy intake and differences in energy intake with age [19]. The data were then categorized into the food groups included in the DASH index and data was converted from grams to servings using values taken from the United States Department of Agriculture (USDA) Food Composition Database [21]. Where available, we used food group data that included disaggregated data from composite dishes (fruit, vegetables, cheese, meat, fish, and legumes). Where this was not available (dairy other than cheese, eggs, sweets, and oils), non-disaggregated data were used [17,22]. We used the data on servings of each food group to generate the DASH index, following the scoring used by Gunther et al. [15]. This index is scored out of a total of 80, with a higher score indicating higher diet quality. Each of the food groups are scored out of 10. Where a higher intake is recommended, the maximum score of 10 was given where the intake met the recommendations and lower intakes scored proportionately. For food groups where DASH favors lower intakes (meat, poultry, fish, eggs, fats, oils, and sweets), a score of 10 was given where the intake met the recommendations, and a score of 0 applied where intakes were double the recommended level. Intakes between these values were scored proportionately. Grains and dairy scores were made up of two parts, each scored out of 5, for total grains and high-fiber grains, and total dairy and low-fat dairy. For further details of the scoring of each food group, see Table S1.

### 2.4. Covariates

Age, sex, and ethnicity of the participants were self-reported by all participants. Given the observed non-linear associations of diet with age, we categorized age according to six age groups: 13–15, 16–18, 19–21, 22–24, 25–27, and 28–30 years. Ethnicity was classified according to five groups. Survey year was classified according to the year of data collection.

Socio-economic class (SEC) of the household reference person was reported by the household reference person. We present summary data on this variable in Table 1. However, given the variation across age-groups, with high proportions of “never worked” in the ages 19–21 age group, as well as the likely change in the meaning of this variable from adolescence (where the parent is frequently the household reference person) to early adulthood (where the participant or their partner is more likely to be the household reference person), we decided not to include SEC as a covariate in our analyses.

### 2.5. Statistical Analyses

All the analyses were performed using STATA version 14 (StataCorp., College Station, TX, USA). The weights provided with the dataset were applied to account for sampling and response biases.

The total DASH index score, and each DASH index component, were regressed on age category, adjusting for ethnicity and survey year, to improve precision of the estimates. We used the STATA “margins” command to obtain adjusted predictions of DASH index score and DASH index components for each age category and sex at the means of covariates (ethnicity and survey year).

To investigate the variation in intakes of each of the DASH index food group components in more detail, we analyzed the associations between intake of each food group (in grams) with age category. As above, we used the STATA “margins” command to obtain adjusted predictions of mean intake in each age category at the means of covariates. 

## 3. Results

Individuals aged between ages 13 and 30 years who had completed a food diary of at least three days (*n* = 2989) were eligible for inclusion. Of those included in this analysis, 98.2% had completed a food diary over four days. Eight participants were dropped from the analyses due to implausible energy intakes, and two individuals were dropped due to missing covariate data, leaving 2979 individuals for analyses. The sample was weighted to be representative of the U.K. population and the weighted socio-demographic data are presented in Table 1.

**Figure 1:**
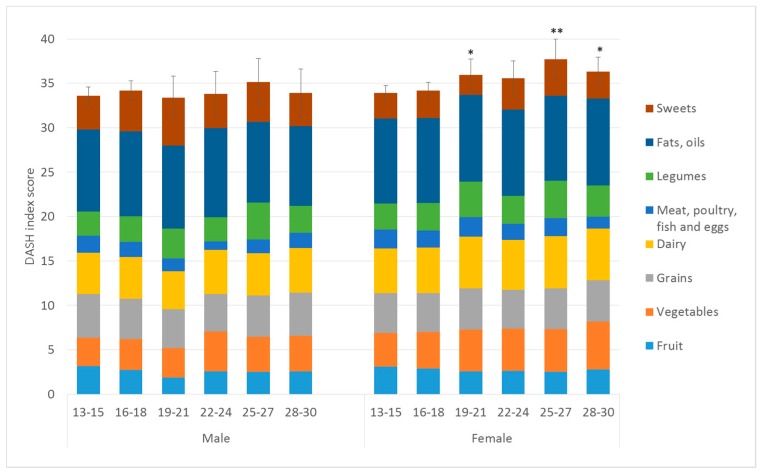
Diet quality (DASH index) by age category and sex for the NDNS Rolling Programme years 1–8. Total DASH scores are out of a maximum of 80 points, with each component score having a maximum of 10 points. Scores were adjusted for ethnicity and survey year. Errors bar indicate a 95% confidence interval of the total DASH score. ** *p* < 0.01, * *p* < 0.05 for the difference between the total DASH score for each age category compared to the reference category (age 13–15).

The mean DASH index score among the population studied was 34.8 (95% CI = 34.3–35.4), out of a maximum score of 80, with 80 representing the highest diet quality. We observed a significant association between sex and DASH index (*β* = 1.58, 95% CI = 0.48–2.68), with higher mean diet quality among females than males. Although we found no statistically significant interaction (*p*-values of *p* = 0.24 and larger) between sex and age category, we report findings of diet quality by age category separately by sex, allowing interrogation of different patterns of the food components that contributed to the diet quality score (Figure 1). After separately analyzing by sex, we saw no significant differences in diet quality with age among males, but a higher diet quality among females aged 19–21 (*β* = 2.04, 95% CI = 0.05–4.02), 25–27 (*β* = 3.77, 95% CI = 1.36–6.18) and 28–30 (*β* = 2.39, 95% CI = 0.53–4.26), compared with ages 13–15. As shown in Figure 1, the score for “meat, poultry, fish, and eggs” was consistently low across the age categories, primarily due to intakes above the recommended values. The fruit score remained low across the age categories and sexes due to low intakes. There was more variation observed in the sweets score, which is reverse-scored such that a higher score represents low sweet consumption (Table S1), with different patterns observed in males and females. While the fats and oils score appeared high (reflecting low intakes), this may be due to lack of inclusion of fats and oils reported as part of composite dishes in our dataset.

Table 2 presents the absolute intake in grams for each of the food group components that make up the DASH index, allowing us to look at variation in levels of consumption with age in more detail. In both sexes, we saw an increase in vegetable intake with age, with greater differences by age category seen in females. In males, we saw a lower dairy consumption at ages 19–21 and 28–30. No association of total dairy intake with age was seen in females, but a higher intake of low-fat dairy was seen in older female age groups. Table 2 also shows a number of food groups where higher or lower intakes were seen in particular age categories, but no consistent trend with age. 

## 4. Discussion

### 4.1. Summary of Main Findings

Our analyses showed limited variation in overall diet quality with age among U.K. adolescents and adults. Diet quality scores remained low at around 35 out of a maximum of 80, with considerable room for improvement seen across almost all component food groups. When the sexes were analyzed separately, small improvements in diet quality were seen among females, but not males, for ages 19–21, 25–27, and 28–30 years in comparison with the 13–15 years age group. More variation in diet with age was observed at the level of the food groups. Among both males and females, fruit intake was lowest at age 19–21, while older participants consumed more vegetables compared to younger participants. Dairy intake was lower among older age categories for males, while among females, there was no change in total dairy intake, but intake of low fat dairy was higher among older age groups.

### 4.2. Comparison with Previous Evidence and Implications of the Findings

The DASH index scores achieved in this population were roughly in line with previously reported scores among other populations. DASH index scores in the NIH-AARP Diet and Health Study ranged from a median of 21.4 in quintile 1 to 43.0 in quintile 5 [9]. Gunther et al. reported mean DASH index scores of 39.9 among youth with Type-1 diabetes mellitus, and 36.6 among youth with type-2 diabetes mellitus [15], a few points higher than the mean scores seen in our study. Our data for food group intakes were similar to those reported from the National Diet and Nutrition Survey, taking into account differences in food group definitions [23]. Nevertheless, these scores were less than half of the maximum score of 80 achieved for a high-quality diet, suggesting much room for improvement in diet quality in our population.

Few studies have focused on the variation in diet quality with age within the adolescent and young adult population. Our findings are consistent with longitudinal findings from the NEXT Plus study in the U.S., which reported small improvements according to two out of three diet quality indices assessed from ages 16 to 20, but did not disaggregate findings by sex [8]. Our overall finding of a higher diet quality among older females is explained by our more detailed findings of higher consumption of vegetables, low-fat dairy, and legumes among these age groups. Greater variation with age might be expected in females than males if these changes are reflective of lifestyle changes, given previous evidence of greater change in dietary intakes across transitions [3] and stronger associations of diet with the home environment [24] among females than males.

Despite the inclusion of fruit juice and potatoes in our definitions of fruit and vegetables, mean intake of fruit and vegetables remained below the recommended intake of five servings per day in almost all age categories. In this analysis we found that vegetable intake did increase with increasing age, while fruit intake was lowest from ages 19–21 and then increased again. The increase in vegetable intakes with increasing age, even through adolescence, is in contrast to previous studies from Brazil and the U.S., which suggest that vegetable intake decreases with age during adolescence [25,26]. In a Norwegian cohort, vegetable intake was observed to decrease up to age 21, before subsequently increasing [3]. It may be that changes in trends over time and differences across cultures are responsible for these different patterns. 

Dairy intake is important in adolescence, associated with improved adolescent cardiometabolic health [14], as well as reduced risk of cardiovascular disease and diabetes later in life [27]. There is some evidence for positive associations with bone health; however, whether such a relationship is likely to be causal is now debated [28]. While a recommended level of dairy intake is not provided in U.K. dietary recommendations, the Dietary Guidelines for Americans recommend an intake of three cup-equivalents of dairy per day (e.g., 720 g of milk) [29]. Our analyses suggest that dairy intake, already well below recommendations, decreases in males beyond the end of adolescence. No corresponding decrease in total dairy intake was seen females, but from age 19 onwards, intake of low-fat dairy begins to increase with age, suggesting that low-fat is replacing full-fat dairy intake. Other studies have shown similar changes in dairy intake during this age range. For example, a study from the U.S. reported decreases in dairy servings among both males and females from age 15 to age 20 [30], while an Australian cohort showed decreases among both males and females from age 14 to age 17 years [31]. These findings suggest that the end of the teenage years may be a particularly important time to promote dairy consumption, with switching to low-fat dairy proposed as a solution for those concerned about high fat intake. 

### 4.3. Strengths and Limitations

In this study we have investigated changes in diet with age through adolescence and early adulthood. However, it is important to note that as a cross-sectional study, this study has the disadvantage that we were not able to follow the same individuals over time to assess within-person change in diet. Nevertheless, this analysis makes use of eight years of very recent data from a large, nationally representative dataset, allowing us to provide a contemporary picture of variation in diet across the age range of interest, and report results that are generalizable to the current U.K. population. 

Diet was assessed using diet diaries by collecting information on all foods and drinks consumed over four days. This method is considered to be one of the more robust methods of assessing diet in free-living individuals, including adolescents [32]. While data are self-reported, use of a comprehensive method of dietary intake allowed us to adjust for total energy consumed in our analyses. This both takes into account mis-reporting of total energy intake and means that diet quality was assessed independently of total levels of consumption. One limitation of our findings is that it is possible that misreporting of different aspects of diet may vary with age, which would bias our findings, however there is currently no evidence available addressing to support this issue. Random measurement error may also vary by age group, although again there is no data to support this; in these analyses increased measurement error would widen the confidence intervals of our reported findings, and reduce the significance of differences seen between age groups. 

We assessed diet quality using a well-recognized measure of diet quality, the DASH index, following previously published methodology. While there are a number of different options for assessing diet quality, the DASH index performs well in comparison with other diet quality scores at predicting health outcomes [10]. DASH is appropriate for use in adolescents, given evidence of associations with adolescent outcomes [12,13] and inclusion of dairy as a positive component of diet quality [14]. This index also does not include moderate levels of alcohol intake as a positive component of the score (as in the Mediterranean Diet Score [10]), which might be particularly inappropriate in adolescents.

## 5. Conclusions

Overall, we find that diet quality, as assessed by the DASH index, is low in U.K. adolescents and young adults, with small increases in diet quality in early adulthood seen among females but not males. Changes in diet with age were seen at the level of the food groups, with some changes, such as increases in vegetable intake, and switching to low-fat dairy, suggesting an opportunity for improvement in diet in early adulthood. Moreover, changes in food group intakes suggested that, across this age range, changes in behavior were taking place, possibly in response to the ongoing changes in environmental and social context, which typically occur during this life stage. Given these ongoing behavioral changes, this life stage may be a key opportunity for intervention to promote improvements in diet, but more evidence is needed to support appropriate policy and intervention development. Further longitudinal research is needed to investigate the modifiable determinants of changes in diet during this age range and understand differences in dietary trajectories among different population groups. 

## Figures and Tables

**Table 1:** Socio-demographic data on the weighted sample by age category from the NDNS Rolling Programme, years 1–8.

		Age Category (Years)						
		13–15	16–18	19–21	22–24	25–27	28–30	Total
		(*n* = 457)	(*n* = 474)	(*n* = 529)	(*n* = 499)	(*n* = 522)	(*n* = 497)	(*n* = 2979)
Sex (%)	Female	51.1	48.2	51.6	48.2	45.2	54.7	50.0
Socio-economic classification (NS-SEC3) of household reference person (%)	Managerial and Professional Occupations	40.2	38.8	23.2	30.6	38.8	38.3	34.8
	Intermediate Occupations	21.3	22.0	22.3	20.3	15.9	20.5	20.3
	Routine and Manual Occupations	33.8	34.1	32.0	39.0	39.8	37.2	36.0
	Never worked and other	4.7	5.2	22.6	10.1	5.6	4.1	8.9
Ethnic group (%)	White	82.0	84.8	82.2	87.2	86.2	86.5	84.8
	Mixed ethnic group	3.0	2.9	6.3	1.8	1.3	2.9	3.0
	Black or Black British	3.6	4.0	2.3	3.5	3.4	1.4	3.0
	Asian or Asian British	9.6	6.4	4.3	7.6	6.0	5.9	6.5
	Any other group	1.7	2.0	4.9	0.0	3.3	3.4	2.6
Current occupational status (%)	Education	100.0	78.6	44.1	17.0	7.0	2.3	40.2
	Employment	0.0	12.7	33.8	64.5	77.8	80.0	45.8
	Not working	0.0	8.7	22.1	18.5	15.2	17.8	14.0

**Table 2:** Intake of DASH index component food groups, by age category, NDNS Rolling Programme years 1–8.

		Mean Intake for Age Category, Adjusted for Ethnicity and Survey Year (95% CI)					
		13–15 (Reference)	16–18	19–21	22–24	25–27	28–30
Males	Fruit (g/day)	175.9 (157.1–194.6)	153.1 (130.2–176.0)	104.0 (73.1–134.9) ***	159.7 (109.9–209.4)	148.6 (102.5–194.7)	153.3 (108.3–198.3)
	Vegetables (g/day)	185.7 (176.2–195.1)	203.1 (191.3–214.8) *	208.1 (182.9–233.2)	235.1 (200.7–269.6) **	220.6 (190.5–250.7) *	229.5 (208.7–250.4) ***
	Grains (g/day)	687.9 (640.1–735.7)	623.9 (583.8–664.1) *	698.9 (548.0–849.8)	674.6 (559.4–789.9)	741.2 (656.9–825.4)	739.8 (648.9–830.7)
	High-fiber grains (g/day)	83.9 (52.4–115.5)	55.4 (42.5–68.3)	68.3 (8.2–128.3)	62.0 (22.2–101.8)	65.5 (38.3–92.7)	86.4 (41.2–131.5)
	Total dairy (g/day)	241.3 (208.8–273.8)	224.6 (199.9–249.4)	157.0 (116.2–197.9) **	208.8 (167.6–250.1)	201.7 (158.1–245.4)	149.8 (121.5–178.0) ***
	Low-fat dairy (g/day)	93.7 (25.7–161.8)	68.3 (45.6–91.0)	135.0 (−16.8–286.8)	95.3 (30.1–160.5)	182.6 (42.6–322.5)	104.8 (41.9–167.8)
	Fish, eggs, meat, and poultry (g/day)	139.3 (131.5–147.0)	156.6 (146.5–166.7) **	176.3 (152.6–200.0) **	165.0 (146.5–183.4) *	179.2 (134.7–223.7)	150.8 (136.5–165.1)
	Nuts, seeds, legumes, and beans (g/day)	13.5 (11.2–15.8)	16.3 (13.1–19.5)	19.6 (11.8–27.3)	13.8 (8.4–19.3)	26.2 (18.2–34.3) **	17.4 (12.1–22.6)
	Oils (g/day)	7.18 (6.13–8.23)	5.97 (4.96–6.99)	6.16 (4.25–8.07)	3.57 (2.23–4.92) ***	7.47 (5.05–9.89)	8.40 (6.13–10.7)
	Sweets (g/day)	43.5 (39.4–47.6)	34.5 (30.7–38.3) **	28.8 (20.6–36.9) **	36.2 (26.9–45.5)	28.5 (21.3–35.7) **	37.8 (29.3–46.4)
Females	Fruit (g/day)	171.7 (157.1–186.4)	160.6 (145.1–176.1)	139.5 (115.0–164.0) *	149.5 (128.2–170.8)	144.5 (117.6–171.3)	160.6 (139.3–182.0)
	Vegetables (g/day)	205.9 (196.9–214.9)	222.3 (212.8–231.8) *	246.8 (226.3–267.3) ***	260.2 (231.9–288.6) ***	251.5 (231.6–271.4) ***	260.2 (242.6–277.9) ***
	Grains (g/day)	604.3 (575.4–633.2)	598.4 (567.0–629.9)	594.3 (534.7–653.8)	625.9 (565.8–685.9)	656.0 (588.2–723.8)	721.5 (642.8–800.1) **
	High-fiber grains (g/day)	47.9 (38.7–57.0)	56.1 (45.0–67.1)	60.5 (35.8–85.1)	67.7 (36.5–98.9)	66.9 (44.3–89.6)	63.9 (44.6–83.1)
	Total dairy (g/day)	174.4 (160.1–188.7)	167.7 (155.1–180.2)	187.4 (162.4–212.5)	171.7 (145.5–197.8)	192.5 (165.4–219.6)	180.8 (162.4–199.1)
	Low-fat dairy (g/day)	77.6 (50.6–104.6)	75.9 (59.3–92.4)	134.4 (75.0–193.7)	152.3 (56.2–248.4)	138.6 (94.0–183.3) *	141.5 (90.0–193.0) *
	Fish, eggs, meat, and poultry (g/day)	129.1 (123.3–134.9)	134.3 (128.5–140.2)	138.0 (121.3–154.8)	147.8 (134.2–161.4) *	139.2 (125.4–152.9)	164.0 (150.7–177.3) ***
	Nuts, seeds, legumes, and beans (g/day)	14.5 (12.2–16.8)	14.7 (12.9–16.6)	22.5 (16.8–28.3) *	15.5 (11.2–19.7)	21.2 (16.9–25.4) **	15.9 (12.5–19.3)
	Oils (g/day)	6.94 (6.23–7.66)	6.65 (5.88–7.42)	5.55 (4.20–6.90)	6.63 (5.32–7.95)	7.49 (6.22–8.76)	6.57 (5.46–7.68)
	Sweets (g/day)	43.1 (39.1–47.2)	40.3 (36.3–44.2)	42.2 (36.1–48.2)	34.8 (27.4–42.1)	37.4 (29.3–45.5)	41.2 (35.2–47.2)

*** *p* < 0.001, ** *p* < 0.01, * *p* < 0.05, regression of food group on age category with age 13–15 as reference category. In line with food group definitions for the DASH index score, fruit includes fruit juice, and vegetables include potatoes.

## Supplementary Materials

The following are available online at http://www.mdpi.com/2072-6643/10/11/1585/s1, Table S1: Scoring of DASH index.
