# Supplementary material for: Diet Quality through Adolescence and Early Adulthood: Cross-Sectional Associations of the Dietary Approaches to Stop Hypertension Diet Index and Component Food Groups with Age
Source: Nutrients. 2018 Oct 29;10(11):1585. doi: 10.3390/nu10111585 (PMC6265851; doi:10.3390/nu10111585)
Supplement: Supplementary file 1 [file nutrients-10-01585-s001.pdf]

Table S1. Scoring of the DASH index score.

Score Component	Maximum Score	Requirement for Maximum Score	Requirement for Minimum Score (0)
Fruit [1]	10	≥4 servings/day	0 servings/day
Vegetables [2]	10	≥4 servings/day	0 servings/day
Total Grains	5	≥6 servings/day	0 servings/day
High-fiber grains	5	≥50% daily grains servings	0% daily grains servings
Total Dairy	5	≥2 servings/day	0 servings/day
Low-fat Dairy	5	≥75% daily dairy servings	0% daily dairy servings
Meat, poultry, fish, and eggs	10	≤2 servings/day	≥4 servings/day
Nuts, seeds, legumes, and beans	10	≥4 servings/week	0 servings/week
Fats and oils	10	≤3 servings/day	≥6 servings/day
Sweets	10	≤5 servings/week	≥10 servings/week
**Maximum total score**	80		

Data are for 2000 kcal/day. Intakes between minimum and maximum levels were scored proportionally. [1] Fruit includes fruit juice. [2] Vegetables include potatoes.
